# Sex-Specific Immune Responses Mediate Host Specificity in Hookworm Infections

**DOI:** 10.3390/tropicalmed10030060

**Published:** 2025-02-25

**Authors:** Andrea Langeland, Catherine A. Jackson, Elise L. McKean, Hajar Errahmani, Damien M. O’Halloran, John M. Hawdon

**Affiliations:** 1 Department of Microbiology, Immunology, and Tropical Medicine, The George Washington University, Washington, DC 20052, USA; catherine.jackson@gwu.edu (C.A.J.); elisemckean@gwu.edu (E.L.M.); herrahmani02@gwu.edu (H.E.); 2 Department of Biological Sciences, The George Washington University, Washington, DC 20052, USA; damienoh@gwu.edu

**Keywords:** host specificity, hookworm infections, *Ancylostoma ceylanicum*, *Ancylostoma caninum*, mouse models, *Stat6* pathway, innate immunity

## Abstract

Hookworm infections affect 500–700 million people worldwide and can lead to chronic conditions, such as malnutrition and anemia. The challenge of managing these infections is heightened by the absence of effective vaccines and the potential for anthelmintic resistance to develop. A comprehensive understanding of the molecular interactions between the parasite and host is vital for unraveling the complexities of infection dynamics. This study aimed to identify the immune system components responsible for host specificity in hookworms by infecting immunodeficient mouse models. Findings herein indicate that innate immunity is essential in protecting against *Ancylostoma ceylanicum* establishment in mice. Significant differences in parasite development were noted in mice lacking the signal transducer and activator of transcription 6 (*Stat6*^−^), with female mice reliant on this Th2 pathway for protection. Secondary infections in female *Stat6*^−^ mice and an immunodeficient NSG mouse reached patency, demonstrating that immunodeficient mice fail to develop protective immunity against subsequent infections, similar to human hookworm infections. In contrast, no parasite development was observed in mice infected with *A. caninum*, highlighting that the survival strategies of this species are independent of the host immune landscape. These results underscore the complexity of host–parasite interactions and point to new directions for therapeutic strategies, which may differ between sex.

## 1. Introduction

Hookworms are soil-transmitted nematodes (STNs) that impose a significant global health burden, affecting 500–700 million people worldwide [1]. These blood-feeding intestinal worms can lead to debilitating and sometimes fatal iron deficiency anemia [2,3]. Infections are particularly devastating in children, resulting in stunted physical and cognitive development, which may be permanent [4,5]. Both pregnant women and the elderly are at a high risk for morbidity, with pregnant women facing risks, such as low birth weight and increased infant mortality [6]. The substantial health impacts of hookworms highlight the urgent need for a better understanding of hookworm infections to develop more effective control strategies and interventions.

To address the health burden posed by hookworms, it is essential to understand the molecular mechanisms driving host specificity, which varies significantly across different host–parasite associations. Parasites range from strict specialists capable of infecting only one or a few genetically similar host species to generalists that can exploit numerous host taxa [7]. Hookworms cover the entire spectrum, from strict specialists, like *Necator americanus*, capable of developing only in humans, to broad generalists, like *Ancylostoma ceylanicum* that can exploit several hosts species. Although hookworms as a group can infect a diverse range of hosts, the factors determining their individual host specificity are poorly understood. Exposing the molecular determinants of host specificity could lead to significant breakthroughs in understanding parasite biology and identifying novel intervention targets.

While *A. ceylanicum* can parasitize multiple species, including humans, dogs, cats, and hamsters, it notably does not establish in mice. However, studies have shown that a small fraction of the infective dose can resume development within the first 3 days in outbred mice, but they fail to establish and are expelled by day 8 post-infection (PI) [8]. Yet, when mice are immunosuppressed with hydrocortisone acetate, *A. ceylanicum* infective third-stage larvae (iL3s) were able to mature into gravid adults, suggesting that hookworm development may be regulated by the host immune response. Furthermore, oral infection with adult hookworms leads to attachment and feeding in various mouse strains, causing anemia and weight loss before the parasites are expelled by day 14 PI [9]. This suggests that structural differences in the small intestine between permissive and non-permissive hosts do not hinder attachment and feeding. In a permissive host, conditions support parasite development and allow for the completion of its life cycle. In contrast, a non-permissive host prevents development and may expel the L3s or drive them into hypobiosis, where they remain infective until a permissive host becomes accessible [10].

Our previous work has demonstrated that immune mechanisms dictate host specificity in the generalist *A. ceylanicum* but not in the specialist *A. caninum* [11]. Additionally, our comparative transcriptomic analyses of intestinal cells from permissive hamsters and non-permissive mouse hosts have revealed distinct immune responses between these two hosts [12]. Notably, the Th2 immune pathway was significantly enhanced in non-permissive mice, highlighted by the upregulation of interleukin-4 receptor alpha (IL-4Rα) and interleukin-13 receptor alpha 2 (IL-13Rα2). In contrast, IL-4Rα was downregulated in permissive hamsters, while there were no significant changes in IL-13Rα2.

To explore which specific arms of the immune system contribute to protection, we infected signal transducer and activator of transcription 6 (*Stat6*)-knockout mice (*Stat6*^−^) with *A. ceylanicum*. These mice exhibit impaired Th2 immune responses, allowing us to examine the specific role of this pathway in hookworm susceptibility. Our findings indicate a sex-dependent permissiveness in *Stat6*^−^ mice, which prompted further investigation into the role of sex hormones. For instance, estrogen and testosterone are known to modulate immune responses, with estrogen often enhancing immunity and testosterone generally suppressing it [13]. This modulation may contribute to differences in susceptibility to infections between males and females. Observations in *Stat6*^−^ and NOD *scid* mice suggest that innate immune mechanisms are responsible for protection against *A. ceylanicum* establishment. In contrast, *A. caninum* does not appear to rely on the host immune landscape for its establishment and may have evolved unique survival strategies, such as hypobiosis and paratenesis, to optimize transmission within its relatively narrow host range. Understanding the molecular mechanisms that mediate host specificity is critical for developing new, effective intervention strategies against hookworm infections, which, as demonstrated, may vary based on the hookworm species and host sex.

## 2. Materials and Methods

Five different mouse strains were used in this study. NSG mice contain the *scid* mutation in the DNA repair complex protein Prkdc and the complete null allele of the interleukin-2 receptor common gamma chain (*IL2rg*^null^) (JAX stock #005557; The Jackson Lab, Bar Harbor, ME, USA). These mice are completely immunodeficient, lacking mature T and B cells, with defective cytokine signaling that results in a deficiency of natural killer cells [14]. One NOD *scid* gamma (NSG) mouse (kindly donated by the Fernandes Lab) at George Washington University (GW) was reinfected. This mouse had previously remained patent for 121 days during its primary infection. The remaining mice were obtained from the Jackson Laboratory. This included NOD *scid* mice, which possess the same *scid* mutation in the *Prkdc* gene as NSG mice but lack the *IL2rg*^null^ mutation. As a result, they have no functional T or B cells, but retain a normal hematopoietic environment and an intact innate immune system (Jax stock #001303) [15]. *Stat6*^−^ mice (JAX stock #005977) exhibit lymphocytes that do not proliferate in response to IL-4, impairing Th2 immune responses [16]. Homozygous estrogen receptor 1 (*Esr1*)-KO mice have no tissue response to estrogen and estrogen receptor alpha activity (JAX stock #026176) [17]. Finally, C57BL/6NJ (Black 6N) mice were used as a control for *Esr1* KO as they have the same genetic background (JAX stock #005304). Syrian golden hamsters (*Mesocricetus auratus*) were bred in-house at GW and used for *A. ceylanicum* maintenance.

*Ancylostoma ceylanicum* (US National Parasite Collection No. 102954, Washington, DC, USA) was maintained in the Syrian golden hamster, while *A. caninum* (US National Parasite Collection No. 106970) was maintained in beagles, following established protocols [11,18]. L3 inocula were prepared as previously described [11] and administered to the animals via oral gavage. The inocula ranged from 80 to 1000 infective third-stage larvae, depending on the hookworm isolate and animal species being tested. Specifically, mice and hamsters infected with *A. ceylanicum* were administered 140 and 80–100 iL3, respectively. For *A. caninum*, mice were administered either 200 or 1000 iL3. Mice were given a 50 μL inoculum followed by 50 μL of nuclease-free water to ensure residual iL3s were washed from the tubes. For hamsters, an inoculum of 100 μL was delivered, followed by a 100 μL rinse. These volumes are determined by the size and weight of the animals, ensuring safe and effective delivery. On the day of infection, the NOD *scid* mice were ~10 weeks old, the Black 6N mice were ~10–11 weeks old, and the *Esr1* mice were ~10–13 weeks old. The *Stat6*^−^ mice had a broader age range of ~6–16 weeks of age, as they were involved in both primary and secondary infections. The age of the single NSG mouse used for reinfection was unknown.

Pooled fecal samples from infected mice were collected by placing them on wire mesh flooring over moistened ALPHA pads (Shepherd Specialty Papers, Watertown, TN. USA). Coprocultures were established as described previously [12] and incubated at 28 °C for at least 7 days. Subsequently, iL3s were retrieved using a modified Baermann technique [19], washed, and stored in 25 mL tissue culture flasks (Corning Inc., Corning, NY, USA). To track the hookworm infection over time, quantitative egg counts were performed ~2–3 times per week using a modified McMaster method [20], provided that sufficient fecal material (2 g per cage) was available to ensure accurate measurements. These counts were conducted until infections were no longer patent or animals were euthanized to determine worm burdens.

*Stat6*^−^ mice were euthanized on day 21 PI, and the small intestines were carefully excised, placed in Petri dishes containing PBS, and incubated at 37 °C on a slide warmer. The intestines were cut longitudinally to expose the epithelial lining, allowing the worms to naturally detach for up to 60 min before being collected for counting. NOD *scid* mice were dissected at 7 and 11 days PI to ascertain the timing of larval expulsion compared to an immunocompetent host. To investigate whether hypobiosis occurs with *A. caninum* in NOD *scid* and *Stat6*^−^ mice, one NOD *scid* mouse was euthanized at 17 days PI, and two *Stat6*^−^ mice were euthanized at 10 days PI. Their musculoskeletal tissue, excluding the cranium, viscera, limbs, and tail, was carefully cut into fine pieces and placed in Baermann funnels overnight with PBS to extract any arrested larvae from the tissues [19].

To assess the viability of *Stat6*^−^-derived *A. ceylanicum* relative to those derived from NSG and hamster hosts reported previously [11], 3 male hamsters were each infected with 80 *Stat6*^−^-derived *A. ceylanicum* iL3 larvae. Prior to euthanasia on day 21 PI, pooled quantitative fecal egg counts were performed. Following euthanasia, the small intestines of each hamster were carefully removed, placed in PBS buffer at 37 °C, and dissected longitudinally to retrieve and enumerate the adult worms.

To compare worm burdens across groups, a one-way analysis of variance (ANOVA) was performed using the stats package in R (version 4.2.0). ANOVA was selected as it is suitable for comparing the means of more than two independent groups when assumptions of normality and equal variances are reasonably met. Normality was assessed using the Shapiro–Wilk test from the stats package in R, and homogeneity of variances was tested using Levene’s test from the car package (version 3.1.2). The Shapiro–Wilk test confirmed normality for most groups with three observations (*p* > 0.05), except for *Stat6*^−^ males, which exhibited deviations from normality due to the narrow range of values. For groups with only two observations, normality could not be formally tested, and ANOVA was performed under the assumption of approximate normality. Levene’s test indicated no significant differences in variances among groups for either analysis (*p* > 0.05). Post-hoc pairwise comparisons were conducted using Tukey’s Honest Significant Difference test to identify statistically significant differences between groups. Worm burdens were compared in hamsters infected with *A. ceylanicum* derived from *Stat6*^−^, NSG, or hamster hosts, and separately in *Stat6*^−^ and NSG mice infected with *A. ceylanicum*. Statistical significance was assessed at a threshold of *p* < 0.05 for all analyses. Data visualization was performed using GraphPad Prism (version 10), as well as Python (version 3.12.1), with the pandas library (version 2.2.0) for data handling and the matplotlib library (version 3.5.2) for plotting.

## 3. Results

### 3.1. The Innate Immune System Is Responsible for Protection Against Hookworm Infection in Mice

**Figure 1:**
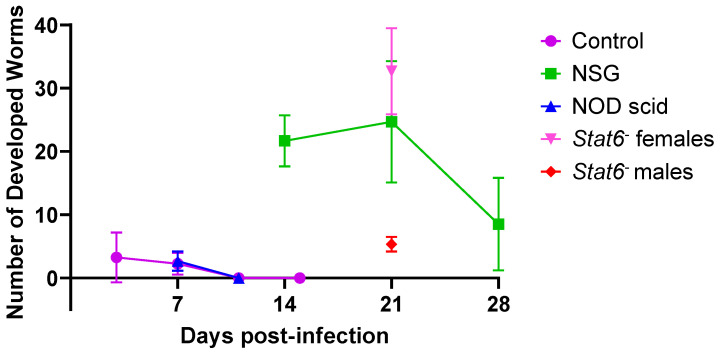
Development of *Ancylostoma ceylanicum* in immunocompetent and immunodeficient mouse strains over time. The x-axis shows the number of days post-infection with approximately 140 *A. ceylanicum* infective larvae, and the y-axis indicates the number of developed worms recovered from the small intestine. Each data point represents the mean worm burden, with error bars indicating the standard deviation for each group. Data for Swiss Webster control mice (purple) and NSG mice (green), previously published [11], are included for comparison. In this study, NOD scid mice (blue) and *Stat6*^−^ female (pink) and male (red) mice each had three biological replicates per time point.

The developmental progression of *A. ceylanicum* in male and female NOD *scid* mice infected with ~140 iL3s revealed that only a few developing worms were present in the small intestine at 7 days PI, and none were detected by 11 days PI. This pattern of infection aligns with previously reported results in non-permissive, immunocompetent Swiss Webster mice (Figure 1) [11]. Three female *Stat6*^−^ mice infected with ~140 *A. ceylanicum* iL3s had high worm burdens of 25, 35, and 38 adult worms in the small intestine at day 21 PI, which were not significantly different from the burdens observed in permissive NSG mice (*p* > 0.05) [11]. In contrast, three *Stat6*^−^ male mice infected with the same dose of iL3s contained significantly lower burdens of 6, 6, and 4 adult worms, which were statistically different from those observed in both female *Stat6*^−^ mice (*p* = 0.006) and NSG mice (*p* = 0.030). Notably, the mouse with the smallest burden had only female worms, while the other two mice had worms of both sexes. Together, these findings suggest that permissiveness to hookworm infection is mediated by the innate immune system in mice and may vary by sex.

### 3.2. The Stat6 Pathway Is Responsible for Hookworm Protection in Female Mice

**Figure 2:**
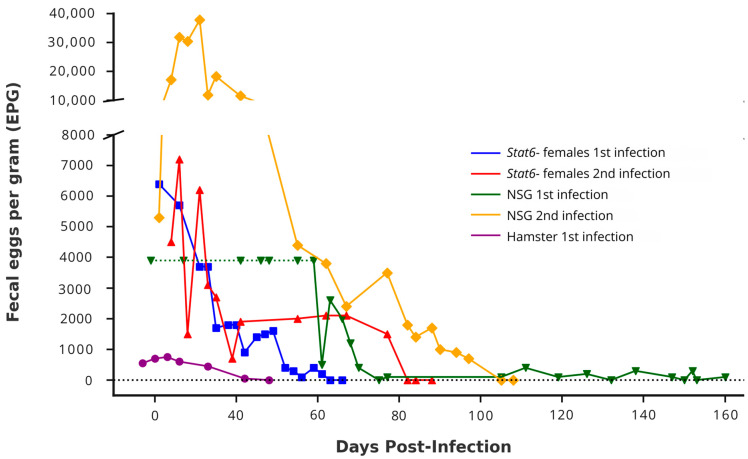
Fecal egg count over time in *Stat6*^−^ and NSG mice following primary and secondary *Ancylostoma ceylanicum* infections. Fecal pellets were collected on the indicated days post-infection (PI). Parasite egg counts are expressed as fecal eggs per gram (EPG), with values representing pooled counts from cages housing the specified animals. The green horizontal dotted line from 19 to 59 days PI indicates the presence of eggs without quantitative EPG data during the first NSG mouse infection, as previously published [11]. Both *Stat6*^−^ infections had EPG values pooled from two female mice housed in a single cage. For the NSG mice, the first fecal EPG assessment included three individual mice until day 77 PI, followed by a second infection with a single mouse. Hamsters were housed individually, as described by Pan et al., 2019 [21].

Two male and female *Stat6*^−^ mice were infected with ~140 *A. ceylanicum* iL3s and checked for patency beginning on day 21 PI. Pooled egg counts from the females first appeared positive on day 21, reaching a peak of 6400 eggs per gram (EPG) of feces. The infection in the female mice remained patent until day 61 PI (Figure 2). Eggs were never detected in the feces from the infected male mice. Approximately 4 weeks after the female mice became egg-negative, as confirmed by three zero EPG readings over the span of 4 days, two female and two male mice were reinfected with ~140 *A. ceylanicum* iL3s. During this secondary infection, only the females became patent. The pooled EPG from the cage housing the female mice reached 4500 on day 24 PI, peaking at 7200 on day 26 PI. The infection remained patent until day 77 PI, with a final EPG of 1500. It is important to note that the secondary infection data are derived from a different primary infection dataset than the one reported here. Although the original primary infection became patent, the data were excluded due to complications with plastic gavage needles, which resulted in inconsistent delivery of the infectious dose. This prompted a switch to stainless steel needles for the secondary infection, and the primary infection was performed again with a new set of mice to ensure consistency.

Two male and two female NOD scid mice were infected with ~140 *A. ceylanicum* iL3s; however, neither sex developed patent infections. Furthermore, six female Esr1-KO mice and six female Black 6N mice, each infected with ~140 *A. ceylanicum* iL3s, failed to develop patent infections, as confirmed through multiple EPG checks. Of these, three mice from each strain were euthanized on day 21 PI to check for the presence of adult worms, but none were detected. The remaining three mice from each strain continued to be monitored for egg output, without any evidence of patency. Finally, one male NSG mouse was reinfected to assess whether it had developed protection from the primary infection, as our previous findings indicated that naïve NSG mice are permissive. This mouse became patent on day 21 PI with an EPG of 5300, which peaked at 37,800 EPG on day 31 PI, and remained patent until day 97 PI.

These infection outcomes were plotted and visually compared to the progression observed in permissive hamsters infected with 150 *A. ceylanicum* iL3s, as well as NSG mice infected with ~140 *A. ceylanicum* iL3s from previously published data (Figure 2) [11,21]. It is important to note that both the reinfected *Stat6*^−^ female mice and the reinfected NSG mouse were not the same animals reported in the primary infection data; however, they had each been infected and had previously achieved patency.

### 3.3. Infectivity of Stat6^−^-Derived A. ceylanicum Larvae for a Permissive Host

To determine if iL3s derived from *Stat6*^−^ mice were viable and infectious, three male hamsters were infected with 80–100 *Stat6*^−^-derived *A. ceylanicum* iL3s, and their infectivity was compared to that of NSG- and hamster-derived *A. ceylanicum* as established previously [11]. Adult worm burdens in the *Stat6*^−^-derived group were 48, 51, and 75 (mean 58.0 ± 14.8 SD) (Table 1). A statistical analysis using ANOVA revealed no statistically significant differences in worm burdens among the three groups (*Stat6*^−^-, NSG-, and hamster-derived *A. ceylanicum*) (*p* > 0.05). These findings suggest that the infectivity of *Stat6*^−^-derived larvae is comparable to that of NSG- and hamster-derived larvae. The pooled EPG for the three hamsters infected with *Stat6*^−^-derived larvae was 533, resulting in an average EPG of 178 per hamster. For the two hamsters infected with NSG-derived larvae, the pooled EPG was 4100, with an average of 2050 per hamster. Meanwhile, the two hamsters infected with hamster-derived larvae had a pooled EPG of 1500, averaging 750 per hamster. Notably, the fecal output from the hamsters infected with *Stat6*^−^-derived worms was lower (1.5 g instead of 2 g), which may have influenced the calculated EPG due to the smaller sample size. Additionally, as this measurement was on the first day of expected egg output, some variability in EPG values is anticipated. However, these EPG values confirm that all groups had reached patency by 21 days PI.

### 3.4. Ancylostoma caninum Fails to Develop in Immunodeficient Stat6^−^ and NOD Scid Mice

To investigate whether the ability to develop in immunodeficient mouse strains differs between *A. ceylanicum* and *A. caninum*, as previously observed in NSG mice, two NOD *scid* mice were infected with ~1000 *A. caninum* iL3s, and four *Stat6*^−^ mice were infected with ~200 *A. caninum* iL3s. In the two male NOD *scid* mice infected with *A. caninum*, one L3 larva was found in the small intestine on day 7 PI (Table 2). On day 17 PI, musculoskeletal tissue (8.51 g) of the second mouse was examined for hypobiotic larvae, but none were found. No worms were found in the small intestine of two male and two female *Stat6*- mice on day 10 PI. The musculoskeletal tissue of one male and one female was examined, revealing 2 L3 larvae in the female (8.26 g) and 1 L3 larva in the male (8.51 g) (Table 2). Adult worms were not detected at any time point. This supports the observation that, unlike in *A. ceylanicum* infections, host specificity is not mediated by the immune system in *A. caninum*.

## 4. Discussion

This study examined the development of the generalist *A. ceylanicum* and the specialist *A. caninum* across five different mouse models: three immunodeficient models (*Stat6*^−^ NOD *scid*, and NSG mice), an *Esr1*-KO model, and an immunocompetent control (Black 6N). In *Stat6*^−^ mice, females uniquely supported the full cycle of *A. ceylanicum*, reaching patency by day 21 PI and sustaining patent infections until day 61 PI. In contrast, infections in males resulted in significantly lower worm burdens by day 21 PI and failed to reach patency. In NOD *scid* mice, a few developing larvae were observed in the small intestine at day 7 PI, but none were detected by day 11 PI, indicating that innate immune mechanisms likely contribute to parasite clearance in these mice. Both male and female *Esr1*-KO mice were non-permissive, with no patent infections observed, suggesting that estrogen receptor activity does not influence host permissiveness to *A. ceylanicum*. Additionally, larvae derived from female *Stat6*^−^ mice were viable and infectious to permissive hamsters, confirming that these mice serve as competent hosts for *A. ceylanicum*. In contrast, *A. caninum* did not reach patency or establish successfully in either *Stat6*^−^ or NOD *scid* mice, suggesting that its host specificity may be determined by an unknown factor or factors independent of the host immune landscape.

The sex difference observed in the permissiveness of *Stat6*^−^ mice highlights an intriguing aspect of the host response, consistent with previous reports of sex-specific immune responses to helminth infections. Studies have frequently documented higher hookworm burdens in males [22,23], a pattern that may result from behavioral factors, such as greater exposure, as well as physiological differences in the immune response associated with sex hormones. This trend suggests that hookworms may have evolved to exploit male hosts more effectively, possibly due to hormonal influences on immunity. However, male-biased parasitism is not a universal rule for helminths, as females can exhibit higher prevalence or intensity in certain infections, while in others, no sex difference is observed [24]. These physiological differences are frequently attributed to the impact of hormones on immune functions. Conversely, species such as *A. duodenale* and *A. caninum* have been shown to undergo hypobiosis in females and transmit larvae vertically through milk [10,25]. This may be an adaptive strategy where larvae arrest development and remain infectious, potentially influenced by the host’s hormonal or immune environment. These strategies suggest a sophisticated evolutionary tactic tailored to exploit different physiological conditions within male and female hosts. However, such adaptive mechanisms vary between generalists and specialists, as vertical transmission has not been observed in *A. ceylanicum*, possibly due to its capability to easily exploit new host species without relying on this method.

Sex hormones, including estrogens, progestogens, and androgens, modulate immune responses through their receptors on immune cells, thereby potentially affecting susceptibility to hookworm infections [13]. Experiments in latently infected lactating dogs have shown that the exogenous administration of estrogen and prolactin stimulates the resurgence of larval output in the milk, highlighting the potential for these hormones to trigger the reactivation of hypobiotic larvae [26]. Moreover, both estrogen and prolactin play a role in upregulating transforming growth factor-β2 (TGF-β2), a cytokine linked to immune regulation during pregnancy [27]. The involvement of TGF-β in the activation of *Caenorhabditis elegans* larvae from their dormant dauer stage, along with its notable effects in stimulating canine hookworms, reinforces the theory that TGF-β originating from the host could act as a trigger for the reactivation of dormant larvae [28]. Although estrogen alone does not directly trigger feeding or reactivation in tissue-arrested *A. caninum* larvae, both TGF-β isoforms 1 and 2 have significant stimulatory effects on tissue-arrested larvae [29], highlighting the hormone-regulated pathway’s role in their reactivation. Hence, high levels of estrogen could signal a robust immune environment, leading to unfavorable conditions for hookworm development during early infection, while these same levels may also signal the opportunity for vertical transmission.

Additionally, the findings that glucocorticoid treatment facilitates the development of *A. ceylanicum* iL3s in non-permissive mouse hosts [8], may reflect the underlying influence of estrogen, given that glucocorticoids have been found to inhibit the immune-stimulating properties of estrogen [30,31,32]. This inhibitory action aligns with the broader immunomodulatory role of estrogen. Notably, female mice have shown a greater dependency on *Stat6* for the production of Th2 cytokines and eosinophils following IL-33 treatment compared to their male counterparts [33]. This observation led to the hypothesis that the reliance on *Stat6* in females might be linked to higher levels of estrogen, especially considering that estrogen receptor-alpha (ER-α) signaling has been implicated in amplifying IL-33 release and group 2 innate lymphoid cell (ILC2)-mediated airway inflammation [34]. Estrogen typically acts as a regulator in the immune system, often suppressing Th1-mediated inflammation while enhancing Th2 responses [35]. However, the lack of permissiveness in female *Esr1*-KO mice to hookworm establishment, as observed herein, challenges the direct involvement of estrogen signaling through ER-α in protection against hookworms. This suggests that *Stat6* might still function effectively in mediating immune responses against hookworms in the absence of direct estrogen signaling through ER-α, potentially involving other hormone receptors and their ligands.

It is essential to identify which pathways confer protective immunity in male *Stat6*^−^ mice. This investigation should include exploring alternative mechanisms, such as Th1 responses, which males may rely on more due to the nature of estrogen in driving Th2 responses in females. Additionally, the potential role of Stat5, capable of initiating Th2 polarization independently of Gata3, should be examined to assess its possible protective mechanism in the absence of *Stat6* [36]. These pathways may provide compensatory immune responses in the absence of *Stat6*, a hypothesis that needs to be tested to fully understand the protective immune dynamics in male mice.

Further investigation is also necessary to elucidate the specific components of the *Stat6* pathway responsible for protection, including both upstream and downstream elements, such as IL-4, IL-13, and eosinophil responses. These components have been implicated in promoting worm clearance in studies involving other parasites [37,38]. These studies could lead to targeted interventions that enhance host resistance against hookworm establishment. Insights into the specific cytokine and cellular responses, specifically those governed by the innate immune system, are critical for developing effective treatment strategies. Importantly, understanding the sex-dependent differences in immune responses is essential, as treatments might need to differ between sexes based on these underlying immunological mechanisms.

In this study, no *A. caninum* larvae were found to be arrested in the male NOD *scid* mouse, while two larvae were detected in one female *Stat6*^−^ mouse and one larva in another male *Stat6*^−^ mouse. Research involving other non-permissive mouse models has shown that larvae from *A. braziliense* and *A. tubaeforme* can survive within the anterior somatic musculature of mice following oral infection, and some larvae have even been detected in the brains of these animals [39]. Additionally, *A. caninum* larvae have been found in the head, thorax, and abdomen of both bred and unbred BALB/c mice following subcutaneous infection [40]. Given that the scope of our investigation of hypobiotic properties in musculoskeletal tissue was limited to a single mouse from each strain, plus a relatively low inoculum and the considerably less than quantitative recovery of larvae from dissected tissue, a comprehensive examination of additional tissues, including the brain and other organs, is warranted to fully understand the hypobiotic potential of *A. caninum* across various mouse models, including both immunodeficient and immunocompetent mice.

## 5. Conclusions

This study highlights the critical role of the innate immune system in determining host specificity for *A. ceylanicum* infections. The differences observed in hookworm development between male and female *Stat6*^−^ mice underscore the complexity of host–parasite interactions. In contrast, *A. caninum* did not establish in either *Stat6*^−^ or NOD *scid* mice, with few or no hypobiotic larvae observed, suggesting that its survival strategies do not depend on the host’s immune landscape. These insights can guide the development of targeted strategies to control and treat hookworm infections, thereby helping to alleviate their significant burden on global health.

## Figures and Tables

**Table 1:** Adult worm burden and fecal eggs per gram in hamsters infected with different isolates of *Ancylostoma ceylanicum* larvae.

Hamster	Sex	Infection Isolate	Worm Burden	Mean ± SD	Combined EPG
1	M	*Stat6*^−^ derived *A. cey*	48	58.0 ± 14.8	533
2	M	*Stat6*^−^ derived *A. cey*	51		
3	M	*Stat6*^−^ derived *A. cey*	75		
4	M	NSG derived *A. cey* *	22	31 ± 12.7	4100
5	M	NSG derived *A. cey* *	40		
6	M	Hamster derived *A. cey* *	35	36 ± 1.4	1500
7	M	Hamster derived *A. cey* *	37		

SD: standard deviation. EPG: eggs per gram. * Data from Langeland et al., 2024 [11].

**Table 2:** Infection outcomes of *Ancylostoma caninum* in *Stat6*^−^ and NOD *scid* mice.

Mouse	Strain	Sex	Infective Dose	Days PI	No. L3s in SI	#L3s in MS	MS Weight (g)
1	NOD *scid*	M	1000	7	1	N/D	N/D
2	NOD *scid*	M	1000	17	0	0	8.51
3	*Stat6* ^−^	F	200	10	0	N/D	N/D
4	*Stat6* ^−^	F	200	10	0	2	8.26
5	*Stat6* ^−^	M	200	10	0	N/D	N/D
6	*Stat6* ^−^	M	200	10	0	1	8.51

PI: post-infection, SI: small intestine, MS: musculoskeletal, N/D: not done.

## Data Availability

Data is contained within the article. Further inquiries can be directed to the corresponding authors.

## Abbreviations

The following abbreviations are used in this manuscript:

STAT6	Signal transducer and activator of transcription 6
STN	Soil-transmitted nematode
PI	Post-infection
NSG	NOD scid gamma
Esr	Estrogen receptor 1
iL3	Infective third-stage larvae
EPG	Eggs per gram
Black 6N	C57BL/6NJ
IL	Interleukin
TGF-β	Transforming growth factor-β
MS	Muscoskeletal
PBS	Phosphate buffered saline
